# Assessing the potential for prevention or earlier detection of on-site monitoring findings from randomised controlled trials: Further analyses of findings from the prospective TEMPER triggered monitoring study

**DOI:** 10.1177/1740774520972650

**Published:** 2020-11-24

**Authors:** William J Cragg, Caroline Hurley, Victoria Yorke-Edwards, Sally P Stenning

**Affiliations:** 1 MRC Clinical Trials Unit at UCL, London, UK; 2 Clinical Trials Research Unit, Leeds Institute of Clinical Trials Research, University of Leeds, Leeds, UK; 3 Health Research Board-Trials Methodology Research Network (HRB-TMRN), National University of Ireland, Galway, Ireland

**Keywords:** Monitoring, central monitoring, on-site monitoring, risk-based monitoring, efficient trial conduct

## Abstract

**Background/Aims:**

Clinical trials should be designed and managed to minimise important errors with potential to compromise patient safety or data integrity, employ monitoring practices that detect and correct important errors quickly, and take robust action to prevent repetition. Regulators highlight the use of risk-based monitoring, making greater use of centralised monitoring and reducing reliance on centre visits. The TEMPER study was a prospective evaluation of triggered monitoring (a risk-based monitoring method), whereby centres are prioritised for visits based on central monitoring results. Conducted in three UK-based randomised cancer treatment trials of investigational medicine products with time-to-event outcomes, it found high levels of serious findings at triggered centre visits but also at visits to matched control centres that, based on central monitoring, were not of concern. Here, we report a detailed review of the serious findings from TEMPER centre visits. We sought to identify feasible, centralised processes which might detect or prevent these findings without a centre visit.

**Methods:**

The primary outcome of this study was the proportion of all ‘major’ and ‘critical’ TEMPER centre visit findings theoretically detectable or preventable through a feasible, centralised process. To devise processes, we considered a representative example of each finding type through an internal consensus exercise. This involved (a) agreeing the potential, by some described process, for each finding type to be centrally detected or prevented and (b) agreeing a proposed feasibility score for each proposed process. To further assess feasibility, we ran a consultation exercise, whereby the proposed processes were reviewed and rated for feasibility by invited external trialists.

**Results:**

In TEMPER, 312 major or critical findings were identified at 94 visits. These findings comprised 120 distinct issues, for which we proposed 56 different centralised processes. Following independent review of the feasibility of the proposed processes by 87 consultation respondents across eight different trial stakeholder groups, we conclude that 306/312 (98%) findings could theoretically be prevented or identified centrally. Of the processes deemed feasible, those relating to informed consent could have the most impact. Of processes not currently deemed feasible, those involving use of electronic health records are among those with the largest potential benefit.

**Conclusions:**

This work presents a best-case scenario, where a large majority of monitoring findings were deemed theoretically preventable or detectable by central processes. Caveats include the cost of applying all necessary methods, and the resource implications of enhanced central monitoring for both centre and trials unit staff. Our results will inform future monitoring plans and emphasise the importance of continued critical review of monitoring processes and outcomes to ensure they remain appropriate.

## Introduction

**Figure 1.**
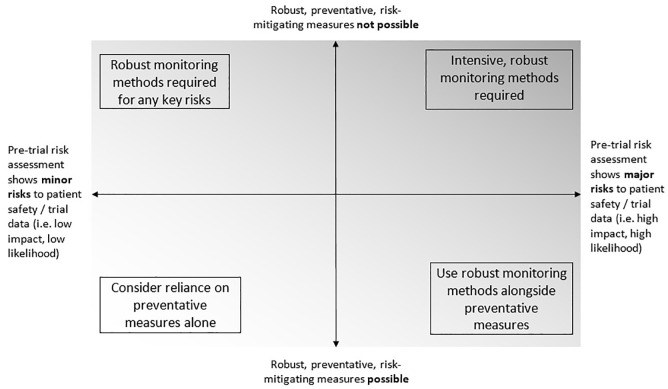
A suggested relationship between preventive measures, monitoring and risk.

A well-run clinical trial is designed and managed to minimise damaging errors in conduct.^1^ Monitoring is done to detect important errors in a reasonable timescale and to enable action to prevent repetition. Cumulatively, this helps ensure the safety of trial participants and the integrity of trial results. Figure 1 shows a suggested relationship between risk, prevention and monitoring.

Historically, and partly in response to regulatory guidance,^2^ trial monitoring has relied on frequent sponsor visits to trial centres.^3^ These visits have clear benefits in terms of building rapport between sponsor representatives and trial centres, delivering training and achieving trial promotion, as well as the opportunity for in-person review of facilities and source documents.^4^ However, on-site monitoring has some significant limitations. The cost of travel and staff time required for regular centre visits is considerable,^5–7^ and may not be justified given the acknowledged limited benefit of source data verification, a common driver of intensive centre visit strategies.^8–10^ Depending on how frequent visits are, on-site monitoring may detect issues less quickly than central monitoring, that is, monitoring conducted without centre visits, using data collected from trial centres. Finally, direct access to individual participant source data, while a strength of on-site monitoring, is less useful than central monitoring when looking for trial-wide issues in multicentre trials.

Acknowledging this, current regulatory guidance now encourages risk-based monitoring^11–13^ with greater emphasis on, and even suggested methods for, central monitoring.^14^ There remains, however, a lack of evidence to support different monitoring practices.^3,15,16^

The TEMPER study (TargetEd Monitoring: Prospective Evaluation and Refinement)^17^ assessed whether centrally monitored threshold-based rules –‘triggers’– could be used as a means to distinguish clinical trial centres with high or low rates of concerning on-site monitoring findings. Three trials participated in TEMPER, each with its own monitoring plan based on prospectively assessed, trial-specific risks. All three trials were phase III randomised cancer treatment trials of investigational medicinal products (IMPs) with time-to-event outcomes. All were already utilising triggered monitoring, as part of their broader monitoring strategy, to prioritise trial centres for visits. Typically, <10% of centres would be visited per year. Compared to trials in other settings (e.g. commercially sponsored trials), there was therefore relatively little source data verification. A typical centre visit might include source data verification for randomly selected five trial participants. In addition to the trigger data review for prioritising centres for visits, central monitoring included regular oversight committee review of summary data on, for example, data return rates^18^ or protocol compliance, and processes to check individual data points for errors or potential concerns with protocol compliance, patient safety or trial integrity.

TEMPER used a prospective matched-pair design to evaluate the effectiveness of the triggered monitoring strategy. Each trial centre prioritised for a visit based on the trigger findings was matched with one from the same trial that was not triggered, that is, not of current concern, and both centres were visited. Centre visits were conducted according to each trial’s monitoring plan, but generally included similar activities across all the trials: review of some or all informed consent forms, source data verification and medical notes review for a sample of participants, facility review (including pharmacy), and review of quality and completeness of essential documents. Findings were categorised in terms of seriousness, according to a standardised system, as Critical, Major or Other, using similar definitions to those of UK regulators.^19^ We considered the collection of Critical and Major findings to be those of interest, that is, ‘errors that matter’.^1^

The full methods and results are reported elsewhere.^17^ TEMPER found that the majority of centres in both triggered visits and matched visits (those without concern) had at least one Major or Critical finding, questioning the efficacy of triggered monitoring as employed in these trials. Here, we report an exploratory review of all the Major and Critical findings reported in TEMPER. We sought to propose centralised monitoring processes or trial process changes which might detect or prevent these findings prior to centre visit. Through this, we aimed to inform and improve future trial conduct by developing an evidence-based (or at least experience-based) central monitoring and quality assurance plan.

## Methods

### Source data: monitoring findings from TEMPER study

**Figure 2.**
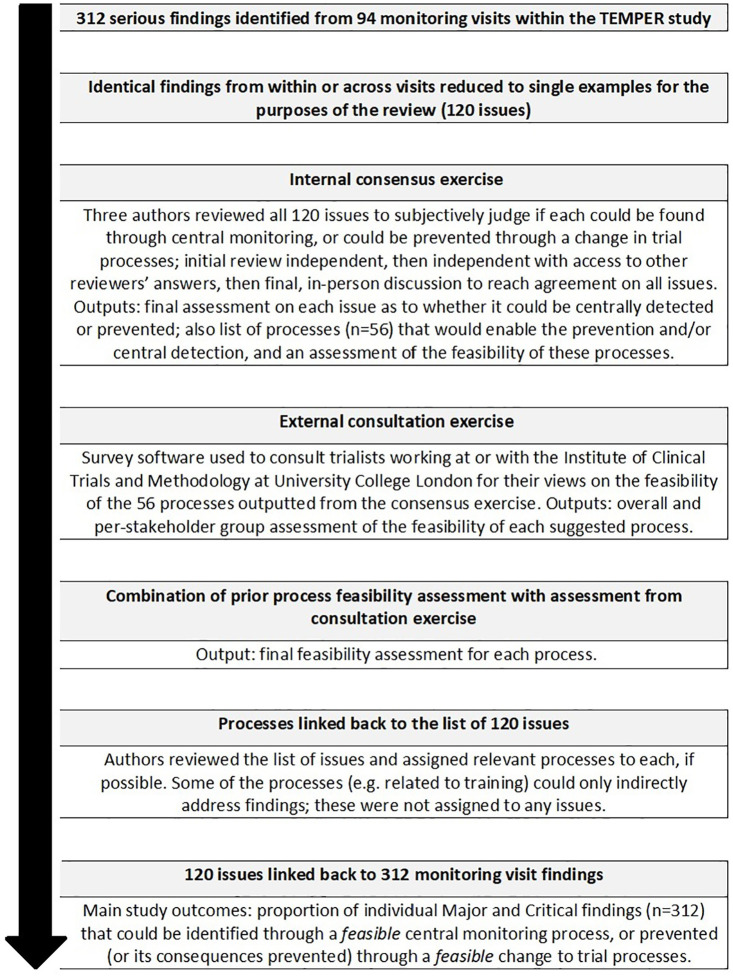
Flow diagram describing this study.

We used findings from all 94 on-site monitoring visits conducted for TEMPER. There were 312 individual Major or Critical findings (298 Major, 14 Critical); these are summarised in Table 1. Some findings had been detected several times within and across centres. In total, there were 120 distinct issues; a representative example of each was reviewed as described below. Figure 2 summarises all the stages of this study.

**Table 1.** Summary of Major and Critical findings at TEMPER monitoring visits.

Type of finding by monitoring report section	Number of findings at all visits (n = 94)			Individual issues^b^
	All findings	Major findings^a^	Critical findings^a^	
Investigator site file – all	6	6	0	5
Informed consent – all	222	219	3	33
Re-consent (e.g. failure to obtain re-consent in a timely manner)	162	162	0	2
Original consent (e.g. missing signatures, missing or in compatible signature dates, incorrect versions used)	60	57	3	31
Pharmacy – all	8	6	2	8
CRF/SDV – all	76	67	9	74
Unreported SAE/notable event	25	25	0	25
Unreported endpoint	12	12	0	10
Source data discrepancy (priority data)	19	19	0	19
Other	20	11	9	20
Total Major and Critical findings	312	298	14	120

CRF: case report form; SDV: source data verification; SAE: serious adverse event.

a The findings reported from the TEMPER study also included some ‘upgrades’ to Major and Critical, that is, groups of findings that collectively indicated a more serious finding. These were not considered relevant outside of the context of the TEMPER study design, and were therefore excluded from this exercise.

b Where a finding had occurred multiple times within or across centres, we reviewed only one of each of these duplications as representative of others; these are listed here as ‘individual issues’ (n = 120).

### Initial review and development of suggested processes (consensus exercise)

Independently of each other, three authors (W.J.C., C.H., S.P.S.) reviewed all 120 issues to consider whether, hypothetically: (1) each issue could be identified through central monitoring and if so, how and (2) the issue could be prevented, or the consequences prevented, through some specified change in trial processes. We considered ‘prevention’ to include both complete prevention of the issue (e.g. process to prevent patient being approached for trial entry if ineligible) and prevention of its consequences (e.g. process to identify erroneously randomised ineligible patient at the time of randomisation and therefore prevent them starting study intervention if not appropriate or safe to do so). Both prevention and central detectability were rated against a five-point scale: 5 = definitely, 4 = possibly, 3 = not sure, 2 = probably not and 1 = definitely not. Each reviewer then reviewed their initial responses alongside those of the other, anonymised reviewers and either confirmed or updated their response for each issue. Finally, reviewers met in-person to agree final results for each issue, using majority votes in cases of any disagreement. The reviewers discussed the potential prevention and central monitoring processes, brainstormed further ideas for these, finalised the process list and agreed a consensus feasibility score for each, using the same five-point scale mentioned above.

### Stakeholder consultation to assess feasibility of suggested processes

We sought independent views on the feasibility of our list of proposed central monitoring and prevention processes through a consultation exercise involving staff at the University College London Institute of Clinical Trials and Methodology, and collaborators working with the Institute’s trials (mainly those working on the three trials involved in TEMPER). We aimed to capture the views of experienced staff both from trial centres (clinicians, research nurses, pharmacists, radiologists) and clinical trials units (trial managers, data managers, trialists and statisticians).

We used Opinio survey software^20^ to develop the consultation exercise. Invitees were not sent any reminders after the initial invite. We asked respondents to review the proposed central monitoring and prevention processes relevant to their role (without disclosing our prior feasibility score) and provide a feasibility score from options ‘Feasible and easy to achieve in current practice’ (score 5), ‘Feasible but expensive or challenging’ (4), ‘Not sure’ (3), ‘Possible but cost or practical issues make it unworkable’ (2) and ‘Not possible at present’ (1). Respondents were given the chance to explain their answers in comments boxes after each process question and by choosing from a pre-defined list of challenges that might apply to each (see Supplementary Files). We also collected information on respondents’ professional role and their experience with clinical trials and with trial monitoring.

We carried out data cleaning prior to analysis. On 15 occasions, respondents had provided free-text comments about the feasibility of particular processes, but no feasibility score; we discussed these and, where we felt the comment was clear, imputed a feasibility score for that response (seven imputations). ‘Not sure’ answers were in one of two groups: either respondents understood the proposed process but were not sure of its feasibility or had said ‘not sure’ to express uncertainty about what the proposed process would entail. Where comments were agreed to unambiguously suggest uncertainty about the nature of the process, we removed the ‘not sure’ response from the final data set. No ethical approval was required for the consultation exercise (confirmed using the Health Research Authority’s decision tool).^21^

### Outcomes and analysis

For each process, we calculated the median feasibility score from the consultation per stakeholder group, and across all respondents. For comparison with our prior feasibility grade, we grouped these into broader categories: broadly feasible (median score ≥4), broadly not feasible (median score ≤2) and not sure (median score 3). Where there was any disagreement in overall or inter-stakeholder medians, we reviewed the consultation data in detail and made a final decision about what broad feasibility category to ascribe. Our general approach was to defer to the views of the stakeholders unless we felt they had not understood our description of the proposed process. When respondents suggested that a process was feasible only in certain conditions, we categorised as ‘feasible with caveats’.

Returning to the original list of 120 distinct issues, we applied the final feasibility scores to any processes that might address each one. From this, we were able to determine our main overall outcomes, namely, the proportion of individual Major and Critical findings (n = 312) that could be identified through a feasible central monitoring process or prevented (or their consequences prevented) through a feasible change to trial processes.

Aside from the survey software, all data collection and analysis took place in Microsoft Excel, and W.J.C. managed the data. Independent checks were carried out by S.P.S. on a 10% sample of findings and proposed processes, respectively, to check feasibility scores pre- and post-consultation.

## Results

In our consensus exercise, we agreed that 114 (95%) of the 120 distinct issues were potentially detectable through central monitoring. A different, mostly overlapping list of 114 (95%) was theoretically preventable through simple changes in trial processes. We proposed 56 processes (or process changes), 43 of which could directly address on-site findings from TEMPER and 13 of which we thought could potentially have an impact, but less directly or without being completely fool-proof, for example, additional training for trial centre staff.

All 56 processes were included in the consultation exercise, which was run between 20 December 2017 and 26 January 2018. Table 2 summarises the stakeholder recipients and responders to each process. 87 people completed the exercise, an overall response rate of 19% of those to whom we sent the survey (n = 450). We excluded 10 additional responses that provided monitoring experience information only, without answers to the remaining questions. Mean number of years’ experience in clinical trials was 11 (range 2–31). Most respondents had not personally conducted on-site monitoring (54%) or central monitoring (59%), and had worked at a centre that had undergone central or on-site monitoring (55%).

**Table 2.** Summary of stakeholder groups invited to participate in the consultation exercise, completion rates, clinical trials experience and number of processes reviewed.

Stakeholder group	Site or clinical trials unit (CTU)	Number distributed to	Number of responses	Completion rate	Mean years worked in clinical trials	Number of processes reviewed
Pharmacy	Site	41	18	44%	7.5	5^a^
Site researchers	Site	49	18	37%	5.0	28^b^
Clinician	Site	207	12	6%	20.5	12
Operations	CTU	66	18	27%	8.5	47^c^
Data management	CTU	33	11	33%	10.0	31^d^
Statistician	CTU	27	5	19%	10.0	13
Senior trialist	CTU	6	3	50%	24.0	20
Radiologist	Site	21	2	10%	20.0	23
Total		450	87	19%	11.0	

a Split into two surveys to same invitees, four in one, one in other (one process missed from initial survey in error).

b Site split into two surveys, each to half of invitees, with 14 processes in each.

c Operations split into two surveys, each to half of invitees, with 24 and 23 processes, respectively.

d Data management split into two surveys, each to half of invitees, with 16 and 15 processes, respectively.

After the consensus exercise and stakeholder consultation, the 56 processes were divided into: 43 processes feasible or feasible with a caveat based on the consultation feedback (77%), five not feasible (9%) and eight uncertain (14%). Table 3 shows the top five most potentially impactful processes, in terms of the proportion of all TEMPER’s Major and Critical visit findings that each addresses, first for processes agreed to be feasible or feasible with a caveat, then those agreed not feasible or of uncertain feasibility. All of the top five feasible or feasible-with-caveat processes relate to informed consent, reflecting the large proportion of TEMPER visit findings relating to this aspect of trial conduct. The top five non-feasible or uncertain-feasibility processes comprise three relating to the use of electronic health record data, either accessed directly from trial hospital systems or via nationwide services such as the National Cancer Registration and Analysis Service (NCRAS) in the United Kingdom. One is a suggestion that trial randomisation be prevented until patient eligibility has been confirmed against source data, and the final suggestion is that site-specific essential documentation be held in a centrally accessible location to assist with sponsor monitoring. Common reasons for uncertainty or non-feasibility of these suggestions included information governance challenges, carrying out these processes in short timescales, privacy concerns, and uncertainty about the suitability of electronic health record data for more complex central monitoring tasks.

**Table 3.** The most potentially impactful processes^a^ agreed feasible or feasible with caveats, and those agreed not currently feasible or of uncertain feasibility.

Process description	Feasibility rating	Finding type addressed	Number of visit findings addressed	% of all visit findings	% of total finding type^b^	Additional information summarised from consultation exercise: (a) for feasible processes: caveats, if applicable; (b) for non-feasible processes: reasons not feasible or not sure
Top five most potentially impactful processes agreed to be feasible or feasible if adjusted in specific ways						
Sites to complete logs to record when patients have re-consented to updated trial information	Feasible	Informed consent	163	52%	73%	N/A
Sponsor to specify a deadline for patients to re-consent to updated trial information, and to chase up any re-consents not done as a matter of urgency	Feasible	Informed consent	163	52%	73%	N/A
Sponsor to distribute (via sites) a letter about updated trial information as well as, or in some cases instead of, a signed re-consent process, so that patients definitely have a chance to be informed within a short timeframe (i.e. not just waiting until the next trial visit)	Feasible with adjustments to process	Informed consent	163	52%	73%	Caveats: approach needs to be ethically approved, expectation that sponsor could cover postage costs, suggest only used for urgent updates. Could be sent directly to participants for CTUs that have direct participant contact.
Central review of completed consent forms prior to randomisation (randomisation cannot proceed without this check)	Feasible with adjustments to process	Informed consent	62	20%	28%	Caveat: not possible in trials with short lead-up time before randomisation.
Central review of completed consent forms at some point after randomisation (so randomisation can proceed without this check)	Feasible	Informed consent	60	19%	27%	N/A
Top five most potentially impactful processes agreed after the consensus exercise to be not feasible or of uncertain feasibility						
Prevent randomisation until all key CRF data have been validated against centrally collected source data (e.g. blood test results sent to sponsor and used to validate CRF data prior to randomisation)	Not sure	Case report form/source data verification	26	8%	34%	Uncertainty due to: potential to be burdensome, challenges achieving this in short timescales, information governance and privacy concerns, possibility of clinical expertise to be required for central review.
Central (Sponsor) access to hospital electronic records for source data verification or other processes	Not feasible	Case report form/source data verification	25	8%	33%	Not feasible due to: information governance and privacy concerns, logistical issues.
Using national databases to look for signs of unreported serious adverse events (e.g. Hospital Episode Statistics^c^ to look for inpatient admissions)	Not sure	Case report form/source data verification	24	8%	32%	Uncertainty due to: cost, issues with timeliness of data availability, possible unreliability of data linkage, possible unreliability of data for this purpose.
Investigator site file documents to be held electronically on a system accessible to the Sponsor so the Sponsor can centrally check the site has correct essential documents	Not sure	Other	7	2%		Uncertainty due to: lack of clarity around whether technology exists to support this, whether contractual agreements required to support this might be difficult to set up, cost, information governance, difficult validation requirements.
Using national databases (e.g. cancer registry data such as NCRAS^d^) to identify unreported disease progression	Not sure	Case report form/source data verification	3	1%	4%	Uncertainty due to: lack of experience doing this, cost and time required, information governance issues, challenges in using personal data under new data protection laws, difficulties applying process to international trials, uncertainty about suitability of data for this purpose, issues of timeliness of data availability.

N/A: not applicable.

a Excluding processes (n = 13) that could potentially have an impact, but less directly or without being completely fool-proof, for example, additional training for trial centre staff.

b Only for informed consent, and case report form/source data verification.

c https://digital.nhs.uk/data-and-information/data-tools-and-services/data-services/hospital-episode-statistics.

d National Cancer Registration and Analysis Service, http://www.ncin.org/about_ncin/.

Further detail on these results is provided in the Online Tables. Online Table S1(a) lists all the processes deemed feasible, and the number and proportion of on-site monitoring findings each addresses; Online Table S1(b) lists all the processes deemed not currently feasible or of uncertain feasibility, with summarised reasoning for each judgement. Online Table S1(c) summarises the feasibility of the indirect or not fool-proof processes.

Based on the consultation exercise, 304/312 (97%) TEMPER visit findings could be detected through feasible central monitoring methods, and 260/312 (83%) were theoretically preventable, or their consequences preventable, through feasible changes to trial processes. 306/312 (98%) were either centrally detectable, preventable, or both. 256/312 (82%) findings were addressed by more than one suggested process or process change, although this varied across the types of finding (informed consent findings: 222/222, 100%; source data review findings: 21/71, 30%).

Table 4 lists abridged summaries of the six remaining findings (three each from triggered visits and non-triggered, matched control centre visits) that could not be centrally detected, or prevented, by a feasible process. All were classified Major; no Critical findings remained. All were from case report form checks and source data verification, with no findings remaining from pharmacy, essential document or informed consent form checks. As with the TEMPER study findings as a whole,^17^ there is no indication that these findings would have had any significant impact on the results or interpretation of the trials involved.

**Table 4.** On-site monitoring findings judged not centrally detectable or preventable, following consensus exercise on feasibility of suggested processes.

Abridged summary of finding	Number of instances	Grading	Reasons why not centrally detectable or preventable
Incorrectly graded adverse event – reported as CTCAE grade 2 instead of 4 (life-threatening); no serious adverse event reported	1	Major	No simple way to detect this issue without review of source data; if serious adverse event had been submitted, could have cross-checked with those data. Currently not possible to access and use hospital episode statistics or other electronic health records to detect this. No obvious fool-proof way to prevent.
Unreported serious adverse event; considered ‘notable event’ in this trial and case report form contained specific question about whether this type of adverse event had occurred, to which answer given had been ‘No’	2	Major	Method to centrally identify cases did not work because centre misreported; therefore, only way to detect is through review of source data. Currently not possible to access and use hospital episode statistics or other electronic health records to detect this. No obvious fool-proof way to prevent.
Unreported serious adverse event due to prolongation of hospital stay	1	Major	Prolongation of hospital stay may only be clear from detailed review of medical notes. Currently not possible to access and use hospital episode statistics or other electronic health records to detect this. No obvious fool-proof way to prevent.
Misreporting of concomitant medication use at randomisation (key baseline data in this trial)	2	Major	Data misreported; not feasible to request source data about concomitant medication to be sent to trials unit for verification; therefore; only way to detect is through on-site monitoring. No obvious fool-proof way to prevent.

## Discussion

We found that a large majority of important on-site monitoring findings – including all categorised as ‘Critical’– could theoretically be detected through feasible central monitoring processes or prevented altogether through feasible changes to trial processes. These results corroborate those of the previous, similar work in a different setting.^22^ A large number of findings, especially in informed consent monitoring, could be addressed by more than one process. Exclusion of the 306 ‘preventable’ or ‘centrally detectable’ findings reduces the number of TEMPER monitoring visits with ≥1 Major or Critical finding from 81/94, as in the primary TEMPER study results, to just six (three at triggered visits, three at untriggered). Our results support the wider trend of replacing some traditionally on-site monitoring activities with centralised activity. However, alongside this shift, there must be targeting of central monitoring efforts, as is implied by monitoring being ‘risk-based’: this means targeting towards errors that matter, including those that are known to occur and, of those, the ones known to occur *most frequently.*

It is important to note that this represents a best-case scenario. Some of the processes will already be in place for some randomised controlled trials (RCTs), including to some degree in the TEMPER trials. We cannot, therefore, easily say how many potential on-site monitoring findings were already successfully avoided through use of these processes in these trials. We did not aim to prove that the TEMPER on-site monitoring findings would definitely have been found earlier through our suggested processes, only to use the findings to develop a more comprehensive plan for future trials. Nonetheless, this may imply that, as currently used, some processes may be insufficient to prevent or centrally identify all issues, or that they cannot be – or have not so far been – implemented consistently across centres and time. Furthermore, an unavoidable limitation of many central monitoring processes is their reliance on good quality and timely reporting from centres.

An increased focus on central rather than on-site monitoring may have implications for resourcing, and we acknowledge that it was beyond the scope of this work to add new quantitative data on this. However, from free-text responses to our consultation exercise (data not shown), it is clear some centre research staff already feel that some central monitoring processes make large demands of their time, without adequate resourcing or recognition (as noted by others).^23^ Central monitoring also displaces resource within the trials unit from dedicated on-site monitors towards the database programmers and statisticians responsible for developing reports and reviewing data, and trial management staff responsible for following up highlighted issues.^24^ Resolving these resourcing questions, perhaps partly through different financial arrangements with centres in trials that rely more on central monitoring, could be a necessary precursor to a more widespread adoption of central monitoring methods.

Our Major and Critical findings were dominated by errors relating to informed consent, because of (1) the importance of consent forms in clinical trial legislation, and therefore also in our monitoring plans and findings categorisation scheme, (2) the relative frequency of errors (not apparently atypical^15,25,26^), (3) the monitoring approach: only one of the three TEMPER trials centrally monitored consent forms, and then not as a pre-randomisation check, and (4) the relatively frequent re-consent requests in these trials, as reported in the main TEMPER report.^17^ We believe (with the support of our consultation) all our findings relating to initial informed consent forms are detectable centrally, at the point of randomisation, thereby preventing patients starting treatment if there are any issues. We suggest that this is a key (if not *the* key) take-home message of this work. By the time of TEMPER’s main publication in 2018, a number of RCTs at the University College London Institute of Clinical Trials and Methodology had started to carry out routine, pre-randomisation central consent form monitoring, whereby a copy of the completed form is checked at the trials unit before being destroyed securely. This may be less feasible in trials with short lead-up times, such as some trials in emergency medicine. Future developments in electronically documenting consent^27,28^ may be useful in resolving many issues we currently face with paper-based forms.

Of the remaining findings, a large proportion related to patient eligibility (recognised as a problem in other trials),^29^ unreported serious adverse events and unreported time-to-event endpoints (in these RCTs, death or cancer progression). Processes to check eligibility prior to randomisation include collection of more detailed data on trial forms to verify eligibility (e.g. blood results) rather than just tick-box confirmation that a patient meets each eligibility criterion, and collecting pseudonymised copies of source data to verify key aspects of eligibility. Our consultation confirmed that both of these are possible, although perhaps not practical in all trials. Some consultation respondents also voiced concern about the availability of clinical expertise required to review medical data before randomisation; it may therefore be best to limit this to objective assessments (e.g. checking blood results are within range) rather than more complex assessments of, for example, scan reports.

Timely reporting of serious adverse events is fundamental to adhering to regulatory requirements for RCTs, and sponsors must have processes in place to ensure all events are received from trial centres. Our consultation exercise reported uncertainty about the current possibility and timeliness of using routinely collected health data in the United Kingdom (e.g. Health Episode Statistics data)^30^ to identify unreported events. For now, our best suggestion is therefore to collect regular data to help ascertain if any serious adverse events may have taken place, such as whether there have been any inpatient hospitalisations or additional treatments since the last follow-up visit. We also suggest that better informing trial participants about safety reporting requirements, particularly where emergency admissions may take place at a non-trial hospital, may improve reporting rates.

Unreported deaths in trials with survival-based outcome measures can be relatively simple to detect centrally by, for example, closely following-up cases where there is no data at the trials unit about a given patient for a long time. It is also feasible to obtain data on patient deaths through national registries, although challenges may remain regarding associated costs and the timeliness of available data. Unreported disease progression can be more difficult to centrally detect, depending on how much additional indicative data are collected. There may be scope for using routinely collected health data to look for changes in patient treatment that may indicate disease progression; however, our consultation respondents felt we could not say this was feasible in the United Kingdom at present.

In the list of processes considered not feasible in our consultation, processes involving use of routinely collected health data were among those with the largest *potential* impact on monitoring. Although there are some reported examples of remote access to individual patient records for source data verification,^31–33^ there are currently substantial legal and information governance barriers to this becoming routine, certainly in the United Kingdom. The feasibility of regular, timely access to linked electronic health record data for research purposes is easier to envisage, and could facilitate identification of unreported (serious) adverse events, trial outcome data and verification of, for example, health economics data. The suitability and availability of these data for complex, time-sensitive purposes is yet to be proven, however.^34,35^

We acknowledge several limitations not already mentioned. The central monitoring processes we propose may be most suitable for trials like those included in TEMPER, which were late-phase trials with already-licenced IMPs posing moderate risk to trial participants. A higher degree of reliance on central monitoring may be less appropriate for higher-risk trials. The feasibility of our monitoring and prevention processes was confirmed not through a broad survey of trialists, but through a convenience sample of staff and collaborators, mainly working on trials of cancer treatments, at the University College London Institute of Clinical Trials and Methodology in the United Kingdom. The consultation exercise had a relatively low response rate. Our results may therefore not be generalisable to different settings or different therapeutic areas. We also acknowledge that our list of consulted stakeholders was not exhaustive, and this work may have benefitted from involvement of individuals representing other bodies, such as research ethics committees or regulatory agencies.

We asked our consultation respondents to comment on each suggested process in isolation, and to implement all processes in combination would be a significant undertaking (or in some cases impossible, where they address a similar issue in different, non-complementary ways). This again highlights the importance of properly resourcing all central monitoring activities. Finally, we should acknowledge that we could implement all these suggested processes in a subsequent trial in a different setting and find different, important findings at trial centres. Comparison with our earlier work^22^ supports this to some extent.

We recommend that trialists review the most common errors in their trials and give due consideration to implementing the central processes – including, but not limited to, those reported in this work – that could detect or prevent the majority of serious issues, alongside processes to address additional, trial-specific risks. There is scope for more formal evaluation of our resulting monitoring plan, and generation of additional data on efficacy and costs, both direct and indirect.

In conclusion, we recommend the process we have explored here of systematically using the results of monitoring to induce continual improvement of trial processes should be a routine, ongoing exercise, as it will ultimately lead to more robust data, safer participants and better trials. Standardised, systematic recording of data about clinical trial monitoring may be a necessary precursor to this, to facilitate review and assessment of trends within and across trials.

## Supplemental Material

sj-pdf-1-ctj-10.1177_1740774520972650 – Supplemental material for Assessing the potential for prevention or earlier detection of on-site monitoring findings from randomised controlled trials: Further analyses of findings from the prospective TEMPER triggered monitoring study Supplemental material, sj-pdf-1-ctj-10.1177_1740774520972650 for Assessing the potential for prevention or earlier detection of on-site monitoring findings from randomised controlled trials: Further analyses of findings from the prospective TEMPER triggered monitoring study by William J Cragg, Caroline Hurley, Victoria Yorke-Edwards and Sally P Stenning in Clinical Trials
