# Supplementary material for: Assessing the potential for prevention or earlier detection of on-site monitoring findings from randomised controlled trials: Further analyses of findings from the prospective TEMPER triggered monitoring study
Source: Clin Trials. 2020 Nov 24;18(1):115–26. doi: 10.1177/1740774520972650 (PMC7876652; doi:10.1177/1740774520972650)
Supplement: sj-pdf-1-ctj-10.1177_1740774520972650 – Supplemental material for Assessing the potential for prevention or earlier detection of on-site monitoring findings from randomised controlled trials: Further analyses of findings from the prospective TEMPER triggered monitoring study [file sj-pdf-1-ctj-10.1177_1740774520972650.pdf]

Alternative monitoring processes – [stakeholder group name]

Page 1

Background: we have analysed a set of on-site monitoring findings from multi-centre phase III trials of cancer treatments, to see which of the findings could have been identified through central monitoring instead, or prevented altogether by alternative processes.

What we are asking of you: we would next like your help in assessing the **feasibility** of the alternative processes we have devised.

On the next pages you will see a list of the alternative processes that are relevant to your role. For each one, we would like your general impression of how feasible this is, in current practice. If possible, please also explain your reasoning using the questions that follow each item.

For your role there are **[x]** processes to review, and we anticipate it will take you about **[x]** minutes to look through them all. There are also a few questions about your role and monitoring experience.

Please also note:

- We are not asking about whether these processes would be successful in improving trial conduct, only about their feasibility.
- We acknowledge some of the suggested processes are probably not feasible at present; it would still be useful to have your input on these and to explore why.
- The findings that inspired these processes are all from phase III cancer trials with time-to-event outcome measures (hence the importance of, for example, finding out when disease progression takes place).

What we will do with your answers: all your responses will be anonymous. Responses will be used to inform development of a peer-reviewed publication about our analysis of on-site monitoring findings. We will not refer to or quote any individual responses from this survey.

If you know us and would rather discuss in person, please feel free to get in touch.

- Will Cragg - MRC CTU at UCL (w.cragg@ucl.ac.uk)
- Sally Stenning - MRC CTU at UCL (s.stenning@ucl.ac.uk)
- Caroline Hurley - University College Cork (carolinehurley@ucc.ie)

Page 2 – Introductory questions

1. How many years have you worked in clinical trials? [Free text]
2. What is your job role? [Free text]
3. What is your experience in clinical trial monitoring? Please tick all that apply.
    - o Conducted on-site monitoring
    - o Conducted central monitoring
    - o Work or worked at a site undergoing on-site or central monitoring
    - o None
    - o Other (please specify) [Free text]

Process questions (same format repeated for each process)

- [Process suggestion, followed by feasibility rating – select one only]
    - o Feasible and easy to achieve in current practice
    - o Feasible but expensive or challenging
    - o Not sure
    - o Possible but cost or practical issues make it unworkable
    - o Not possible at present

- If possible, please briefly explain your reasoning: [Free text]

- Which of the following issues could be serious barriers to this process? (Tick all that apply)
    - o Cost
    - o Time for Trials Unit staff
    - o Time for Site Staff
    - o Logistical issues
    - o Legal or regulatory issues
    - o Other

- Any other comment? (Optional) [Free text]

Additional questions (for some surveys only)[1]

Finally, we also identified some findings from on-site monitoring which we could not envisage finding through central monitoring, or preventing through an alternative process.

Please review the situations listed below and think about the following:

- Could Sponsors identify these through central monitoring, rather than on-site monitoring?
- Could Sponsors make any of these less likely to occur through altering the trial processes?

If the answer to either of these questions is Yes, please give details in the box beneath each scenario.

- [Description for each finding]
    - Suggestions (if any): [Free text]

[1] These questions were added to gather additional ideas for monitoring findings that the authors of this work had considered to be either not preventable or not centrally detectable. However, they did not yield any useful additional information and we therefore have not reported the results in the main publication. The questions are nonetheless included here for transparency.

Final page

- Do you have any further comments? [Free text]

Thank you for taking our survey.

Online Table S1a: all processes agreed after the consensus exercise to be feasible or feasible if adjusted in specific ways, and the number and proportion of findings each addresses, overall and by subtype.

Process Description	Feasibility rating	Caveats, if applicable (summarised from consultation exercise)	Finding type addressed	Number of visit findings addressed	% of all visit findings	% of total finding type[2]
Sites to complete logs to record when patients have reconsented to updated trial information.	Feasible	N/a	Informed consent	163	52%	73%
Sponsor to specify a deadline for patients to reconsent to updated trial information, and to chase up any reconsents not done as a matter of urgency	Feasible	N/a	Informed consent	163	52%	73%
Sponsor to distribute (via sites) a letter about updated trial information as well as, or in some cases instead of, a signed reconsent process, so that patients definitely have a chance to be informed within a short timeframe (i.e. not just waiting until the next trial visit)	Feasible with adjustments to process	Caveats: approach needs to be ethically approved, expectation that Sponsor could cover postage costs, suggest only used for urgent updates. Could be sent directly to participants for CTUs that have direct participant contact.	Informed consent	163	52%	73%
Central review of completed consent forms prior to randomisation (randomisation cannot proceed without this check).	Feasible with adjustments to process	Caveat: not possible in trials with short lead-up time before randomisation.	Informed consent	62	20%	28%

[2] Only for Informed Consent, CRF/SDV and Pharmacy.

Process Description	Feasibility rating	Caveats, if applicable (summarised from consultation exercise)	Finding type addressed	Number of visit findings addressed	% of all visit findings	% of total finding type[2]
Central review of completed consent forms at some point after randomisation (so randomisation can proceed without this check).	Feasible	N/a	Informed consent	60	19%	27%
Collection of detailed information about consent form (e.g. date of completion, name of signing clinician, confirmation of correct completion) as mandatory information prior to randomisation.	Feasible with adjustments to process	Caveat: not possible in trials with short lead-up time before randomisation.	Informed consent	60	19%	27%
When important new trial documents are released (e.g. protocol, patient information sheet), Sponsor to prevent sites randomising further patients until they have received local copies (e.g. signed by Principal Investigator or presented on local headed paper)	Feasible with adjustments to process	Caveat: feasible but use with care, i.e. only for key changes to important documents.	Other	30	10%	
Sponsor requires site to complete investigator site file self-assessment on a regular basis (e.g. at least annually)	Feasible	N/a	Other	30	10%	
Central (Sponsor) collection of anonymised source data to verify important baseline data	Feasible with adjustments to process	Caveats: only for key data and only for objective assessments (e.g.to test result values within correct range)	CRF/SDV	26	8%	34%

Process Description	Feasibility rating	Caveats, if applicable (summarised from consultation exercise)	Finding type addressed	Number of visit findings addressed	% of all visit findings	% of total finding type[2]
Collect detailed data on follow-up CRFs to allow Sponsor to ascertain if there have been any Serious Adverse Events (e.g. number of hospital admissions since last visit, etc)	Feasible	N/a	CRF/SDV	16	5%	21%
Baseline CRFs to request all data to allow Sponsor to confirm eligibility of each patient, rather than just asking site to confirm eligibility (via tick boxes or similar)	Feasible with adjustments to process	Caveat: only for objective assessments (e.g.to test result values within correct range) and may not be feasible in fast-recruiting trials.	CRF/SDV	13	4%	17%
Sites to scan important trial documents (e.g. consent forms) to guard against them being lost subsequently.	Feasible with adjustments to process	Caveat: only for key documents.	Other	12	4%	
Prevent randomisation until all key CRF data has been collected and validated (e.g. don't allow randomisation with out-of-range or out-of-date test results)	Feasible	N/a	CRF/SDV	9	3%	12%
Sponsor to urgently chase up sites about cases where a patient has no follow-up data for over a year, to find out if an endpoint may have occurred.	Feasible	N/a	CRF/SDV	9	3%	12%

Process Description	Feasibility rating	Caveats, if applicable (summarised from consultation exercise)	Finding type addressed	Number of visit findings addressed	% of all visit findings	% of total finding type[2]
Using national databases (e.g. Office for National Statistics or similar) to identify unreported patient deaths.	Feasible	N/a	CRF/SDV	9	3%	12%
Principal Investigator to confirm receipt of important new trial documents, e.g. by signing a front page, or signing a bespoke confirmation document	Feasible	N/a	Other	7	2%	
When important new trial documents are released (e.g. protocol, patient information sheet), Sponsor to request local copies (e.g. signed by Principal Investigator or presented on local headed paper)	Feasible	N/a	Other	7	2%	
Remove the requirement for patients to write on the consent form the version and date of the Patient Information Sheet they read, as this is prone to error.	Feasible	N/a	Informed consent	5	2%	2%
Central (Sponsor) collection of pharmacy accountability logs (electronic or paper) to allow central review	Feasible with adjustments to process	Caveat: collection of all may not be feasible, but sampling approach should be (with escalation/additional sampling as required).	Pharmacy	4	1%	50%

Process Description	Feasibility rating	Caveats, if applicable (summarised from consultation exercise)	Finding type addressed	Number of visit findings addressed	% of all visit findings	% of total finding type[2]
Routine cross-checking of reported adverse events and serious adverse events to check all events have been reported correctly	Feasible	N/a	CRF/SDV	4	1%	5%
Use an additional CRF or CRF section to collect data about late-developing adverse events	Feasible	N/a	CRF/SDV	3	1%	4%
Follow-up CRFs to ask detailed questions about disease status, additional treatment received and any other factors that may help identify unreported disease progressions.	Feasible	N/a	CRF/SDV	3	1%	4%
CRFs to collect data to check all protocol-mandated safety processes have been completed, for example additional tests or patient monitoring in response to raised blood test results	Feasible	N/a	CRF/SDV	2	1%	3%
All relevant database validation in place to automatically identify non-valid or unlikely toxicity grades (e.g. grade 1 symptomatic angina)	Feasible with adjustments to process	Caveat: use risk-based approach to focus on important toxicities to avoid overburdening site and CTU with query management.	CRF/SDV	1	<1%	1%

Process Description	Feasibility rating	Caveats, if applicable (summarised from consultation exercise)	Finding type addressed	Number of visit findings addressed	% of all visit findings	% of total finding type[2]
For selected data points, Sponsor to not accept a site response of 'not done', if this is very unlikely to be true for a given type of patient (e.g. for key diagnostic tests)	Feasible with adjustments to process	Caveat: feasible if used sparingly (i.e. for important data only).	CRF/SDV	1	<1%	1%
Central (Sponsor) collection of pharmacy temperature logs (electronic or paper) to allow central review	Feasible with adjustments to process	Caveat: collection of all may not be feasible, but sampling approach should be (with escalation/additional sampling as required).	Pharmacy	1	<1%	13%
Trial CRFs to ask sites to confirm at each patient visit that radiology requirements (e.g. use of contrast in CT scans) have been followed	Feasible	N/a	CRF/SDV	1	<1%	1%
Mechanisms for reporting retention issues to require in-depth detail about each case, to allow Sponsor to easily ascertain what has happened and the possible effects on further data collection (for example, to more easily tell the difference between cases where patients stop follow-up visits and those where they simply stop trial intervention)	Feasible with adjustments to process	Caveat: feasible if proposed process clarified (i.e. to confirm that the extra information could be collected on a CRF).	CRF/SDV	1	<1%	1%

Process Description	Feasibility rating	Caveats, if applicable (summarised from consultation exercise)	Finding type addressed	Number of visit findings addressed	% of all visit findings	% of total finding type[2]
Sponsor to use a detailed centralised procedure to investigate cases of withdrawal or loss to follow-up, and to agree with sites how to proceed in each case; sites to be aware of and expect this process.	Feasible with adjustments to process	Caveat: feasible if details of proposed process clarified (i.e. to confirm that it would just involve regular team case-by-case review of withdrawn and lost-to-follow-up patients).	CRF/SDV	1	<1%	1%
Sites to send copies of paper delegation logs to Sponsor on a regular basis (e.g. every 3 months)	Feasible	N/a	Other	1	<1%	
Sponsor preventing access to key trial systems (trial database, IVRS system) until site members of staff are correctly listed and signed-off on the site delegation log	Feasible	N/a	Other	1	<1%	

Online Table S1b: all processes agreed after the consensus exercise to be not feasible or of uncertain feasibility, and the number and proportion of findings each addresses, overall and by subtype.

Process Description	Feasibility rating	Finding type addressed	Number of visit findings addressed	% of all visit findings	% of total finding type[3]	Reasons not feasible, or not sure (summarised from consultation exercise)
Prevent randomisation until all key CRF data has been validated against centrally collected source data (e.g. blood test results sent to sponsor and used to validate CRF data prior to randomisation)	Not sure	CRF/SDV	26	8%	34%	Uncertainty due to: potential to be burdensome, challenges achieving this in short timescales, information governance and privacy concerns, possibility of clinical expertise to be required for central review.
Central (Sponsor) access to hospital electronic records for source data verification or other processes	Not feasible	CRF/SDV	25	8%	33%	Not feasible due to: information governance and privacy concerns, logistical issues.
Using national databases to look for signs of unreported serious adverse events (e.g. Hospital Episode Statistics to look for inpatient admissions)	Not sure	CRF/SDV	24	8%	32%	Uncertainty due to: cost, issues with timeliness of data availability, possible unreliability of data linkage, possible unreliability of data for this purpose.
Investigator site file documents to be held electronically on a system accessible to the Sponsor so the Sponsor can centrally check the site has correct essential documents	Not sure	Other	7	2%		Uncertainty due to: lack of clarity around whether technology exists to support this, whether contractual agreements required to support this might be difficult to set up, cost, information governance, difficult validation requirements.

[3] Only for Informed Consent, CRF/SDV and Pharmacy.

Process Description	Feasibility rating	Finding type addressed	Number of visit findings addressed	% of all visit findings	% of total finding type[3]	Reasons not feasible, or not sure (summarised from consultation exercise)
Using national databases (e.g. cancer registry data such as NCRAS) to identify unreported disease progression	Not sure	CRF/SDV	3	1%	4%	Uncertainty due to: lack of experience doing this, cost and time required, information governance issues, challenges in using personal data under new data protection laws, difficulties applying process to international trials, uncertainty about suitability of data for this purpose, issues of timeliness of data availability.
Implement a barcoding or similar system to ensure correct bottles are dispensed in a double-blind trial	Not sure	Pharmacy	2	1%	25%	Uncertainty due to: cost and complexity of systems, negative impact on time for dispensing, lack of experience with such systems, challenges with system maintenance, difficulties adopting different systems for each sponsor
Central (Sponsor) access to electronic pharmacy systems to allow central monitoring of any aspects of pharmacy	Not feasible	Pharmacy	2	1%	25%	Not feasible due to: complications in setting up such an arrangement, prevalence of paper-based (rather than electronic) pharmacy systems.
Using national databases to look for unreported second primary cancers	Not sure	CRF/SDV	2	1%	3%	Uncertainty due to: unreliable or heterogeneous data collection across sites, lack of experience doing such a process, difficulties if not prospectively planned (from start of trial), cost and time involved, more difficult in international trials, issues of data timeliness, issues of patient consent, uncertainty around reliability of data for this purpose.

Process Description	Feasibility rating	Finding type addressed	Number of visit findings addressed	% of all visit findings	% of total finding type[3]	Reasons not feasible, or not sure (summarised from consultation exercise)
Sponsor to receive automated, real-time notifications about pharmacy temperature excursions	Not feasible	Pharmacy	1	<1%	13%	Not feasible due to: time and cost, logistical issues, hard to manage frequent notifications, lack of out of hours coverage, need for judgement from pharmacist in given situation, concern about false alarms.
Central (Sponsor) access to radiological 'prescribing' systems (if/where these exist) to allow central monitoring of adherence to protocol radiological requirements	Not feasible	CRF/SDV	1	<1%	1%	Not feasible due to: relevant information not being available in such systems, regulatory or information governance barriers to external access.
Develop and use an electronic delegation log system, so all site staff are authorised by Principal Investigator and Sponsor always has access to the current site staff list	Not feasible	Other	1	<1%		Not feasible due to: predicted issues with account management, challenges in creating regulatory-compliant system, cost required to set it up, concerns about maintaining sponsor oversight, concerns about system maintenance.

Online Table S1c: processes that could address on-site monitoring findings, but indirectly or without being completely fool-proof, with feasibility rating and, where applicable, suggested adjustments to improve feasibility or reasons why infeasible.

Process description Simplified wording	Feasibility rating	Caveats or reasons not feasible (summarised from consultation exercise)
Routine use of central statistical monitoring techniques in order to identify data quality issues early.	Feasible	N/a
Additional training to help site staff collect certain types of data, e.g. how to extract relevant information from histology reports.	Feasible	N/a
Each clinical trial site to receive an on-site monitoring visit early in their trial participation, e.g. within one month of first randomisation	Feasible	N/a
Sponsor to introduce mechanisms to share common on-site or central monitoring findings with all sites, to help improve overall trial conduct	Feasible	N/a
Sponsor to introduce mechanisms to share common on-site or monitoring findings between trials at the Trials Unit, to help improve trial conduct across all trials	Feasible	N/a
Develop and use a risk-based approach to updated information, only requiring signed reconsent when changes are considered significant.	Feasible	N/a
Sponsor to be clearer about how sites are expected to manage issues with retention in, for example, the trial protocol and site training processes.	Feasible	N/a
Develop a method to inform patients about trial safety reporting requirements, for example a leaflet or similar to encourage patients to tell their site about any adverse events	Feasible	N/a

**Process description Simplified wording**	**Feasibility rating**	**Caveats or reasons not feasible (summarised from consultation exercise)**
Sponsor to prioritise any automated database validations that may have patient safety implications, to allow these to be followed up urgently.	Feasible	N/a
Develop and use formal testing processes to check that protocol requirements are feasible prior to a trial starting, and to understand how similar the protocol is to standard practice	Feasible with adjustments to process	Caveat: could be feasible, depending on exactly what the processes were.<br><br>Author comments: we believe this would definitely be feasible, using methods such as setting up independent committees to advise on this (or using existing groups such as Trial Steering Committees), or consulting tools like PRECIS-2.[4]
Mechanisms for reporting retention or follow-up issues (CRFs or other mechanisms) to include more detail about how sites are expected to manage such situations.	Feasible with adjustments to process	Caveat: could be feasible, but some uncertainty among consultation respondents about what this might entail, and about 'overloading' sites with guidance.<br><br>Author comments: as this would likely constitute extra text on a form (e.g. on a Withdrawal CRF), there does not seem a reason why it wouldn't be feasible. It would also likely be similar advice for most trials, so the text could be standardised.
Sponsor to require all site staff to attend sponsor-led training session prior to work on trial	Feasible with adjustments to process	Caveats: feasible, but concerns about workload for trials unit and site, especially if high staff turnover at site, and about getting all staff together simultaneously for a single training session. More feasible if training is available online and 'on-demand'; more feasible to set a minimum number of staff (or minimum set of roles) to undergo training.

[4] Loudon K, Treweek S, Sullivan F, Donnan P, Thorpe KE, Zwarenstein M. The PRECIS-2 tool: designing trials that are fit for purpose. BMJ. 2015 May 8;350:h2147. https://doi.org/10.1136/bmj.h2147

Process description Simplified wording	Feasibility rating	Caveats or reasons not feasible (summarised from consultation exercise)
Measures to increase engagement with radiologists (e.g. requirement for named radiologist among site trial staff, inviting radiologists to investigator meetings etc) in order to increase compliance with protocol requirements regarding radiological imaging.	Not sure	Not considered feasible due to lack of research time available to radiologists.
Sponsor training of site staff to require each staff member to pass a short test on the key points prior to working on the trial	Not sure	Not considered feasible: possibly burdensome for sites, difficult in cases of high staff turnover, and concerns about harming morale and trust in site-CTU relationship.
